# Shape-aware stochastic neighbor embedding for robust data visualisations

**DOI:** 10.1186/s12859-022-05028-8

**Published:** 2022-11-14

**Authors:** Tobias Wängberg, Joanna Tyrcha, Chun-Biu Li

**Affiliations:** Department of Mathematics, Stockholm University, Stockholm, Sweden

**Keywords:** Data visualisation, Dimensionality reduction, Graph distance, Dimensionality reduction validation

## Abstract

**Background:**

The t-distributed Stochastic Neighbor Embedding (t-SNE) algorithm has emerged as one of the leading methods for visualising high-dimensional (HD) data in a wide variety of fields, especially for revealing cluster structure in HD single-cell transcriptomics data. However, t-SNE often fails to correctly represent hierarchical relationships between clusters and creates spurious patterns in the embedding. In this work we generalised t-SNE using shape-aware graph distances to mitigate some of the limitations of the t-SNE. Although many methods have been recently proposed to circumvent the shortcomings of t-SNE, notably Uniform manifold approximation (UMAP) and Potential of heat diffusion for affinity-based transition embedding (PHATE), we see a clear advantage of the proposed graph-based method.

**Results:**

The superior performance of the proposed method is first demonstrated on simulated data, where a significant improvement compared to t-SNE, UMAP and PHATE, based on quantitative validation indices, is observed when visualising imbalanced, nonlinear, continuous and hierarchically structured data. Thereafter the ability of the proposed method compared to the competing methods to create faithfully low-dimensional embeddings is shown on two real-world data sets, the single-cell transcriptomics data and the MNIST image data. In addition, the only hyper-parameter of the method can be automatically chosen in a data-driven way, which is consistently optimal across all test cases in this study.

**Conclusions:**

In this work we show that the proposed shape-aware stochastic neighbor embedding method creates low-dimensional visualisations that robustly and accurately reveal key structures of high-dimensional data.

## Background

Analysing high-dimensional (HD) data is an important challenge in a wide variety of fields. In particular, Dimensionality Reduction (DR) techniques have been increasingly used for visualising high-dimensional data by projecting the data onto a low-dimensional (LD), usually 2D, space. The aim is to reveal the key hidden structures in the HD data, such as clusters or other geometrical arrangements of the data points. One of the most frequently used methods for this purpose is the t-distributed Stochastic Neighbor Embedding (t-SNE) [1]. The t-SNE is able to create compelling visualisations of data with hundreds of dimensions in fields as diverse as image processing [1], speech recognition [2], immuno-profiling of COVID-19 patients [3], etc. One further important area of application is cell biology where data is collected on gene expressions in individual cells [4–7]. Cells are often characterised by expressions of thousands of different genes, where the t-SNE has enabled visual analysis of the data in the LD embedding. One of the main successes of t-SNE is its ability to capture discrete patterns even for data with very high dimensions compared with traditional DR methods [1], such as principal component analysis (PCA) [8], locally linear embedding [9], ISOMAP [10] and Laplacian eigenmaps [11], etc. The approach taken by t-SNE focuses primarily on preserving local structures, usually characterised by Euclidean distances (ED), but not on the global arrangement of points. Despite its merits, several drawbacks of t-SNE have been identified in the literature. Specifically, the t-SNE requires the user to define what is meant by local. This is often difficult to assess in practise and an incorrect notion of locality can result in spurious patterns appearing in the LD embedding. Furthermore, global patterns are important in many cases, but they are not guaranteed to be preserved by the t-SNE.

To alleviate these limitations, we propose to incorporate graph-based distances into the framework of t-SNE. The first step of the method is to construct a graph representing the HD data in a data-driven way by only connecting points in small local neighborhoods. Information about the global structures of the constructed graph are then captured by shape-aware graph distances (the biharmonic distance [12] in this study). In contrast to conventional distance measures, such as the ED, shape-aware graph distances are able to learn the global shapes of the underlying manifold or structure on which the HD data reside. This has an advantage for dimensionality reduction based on distance preservation. For example, if the underlying manifold is a 2D structure embedded in a 3D Euclidean space, then an ED-based algorithm would have to give up global distances to reduce dimension. A DR technique based on a shape-aware distance that respects the curvature of the manifold, on the other hand, can reduce dimension without distorting global structure. We term the t-SNE embedding shape-aware distances SASNE, short for Shape-Aware Stochastic Neighbor Embedding. The original t-SNE that embeds conventional distances, e.g. ED, is hereafter simply referred to as t-SNE.

Some recent methods have also been proposed that claim to solve the shortcomings of t-SNE; in particular the Potential of heat diffusion for affinity-based transition embedding (PHATE) [13] and the Uniform manifold approximation (UMAP) [14, 15] methods. In this study, we compare SASNE to t-SNE, PHATE and UMAP and show that these competing methods are not able to consistently (i) reveal true discrete structures, (ii) avoid creating spurious discrete structures, and (iii) preserve global and hierarchical structures as well as SASNE. In order to confirm the advantages of SASNE compared to t-SNE, PHATE and UMAP, we apply the methods to embed both synthetic and real data sets that demonstrate imbalanced, nonlinear, hierarchical and developmental trajectory structures. The real data sets are the MNIST data set of handwritten digits and gene expressions from cells of a mouse brain.

In some previous works [3–7], judging the embedding performance is often done simply by visual inspection where the embedding quality is assessed by the amount of discrete structures appearing in the LD embedding. However, the discrete structures that appear in the LD embedding may be spurious and do not necessarily reflect the true underlying organisation of the HD data due to, e.g., inappropriate choice of hyper-parameters or failure to capture global structures in the LD space. Here, we scrutinize the embedding quality in terms of quantitative validation methods for both clustering (the silhouette indices and plots) and dimensionality reduction (rank-based methods). It is found that SASNE not only shows significant improvement in preserving both clustering and hierarchical structures at all scales, but also allows us to fix the hyper-parameter of the method in a data-driven way, which is commonly chosen by default [4].

The outline of the paper is as follows. The theoretical concepts of the SASNE are introduced in the “Methods” section. These include an overview of the t-SNE method, the motivation and evaluation of graph-based distances, and the validation methods monitoring the quality of clustering and dimensionality reduction in the LD embedding. In the “Results” section, we demonstrate the superior performances of SASNE in capturing nonlinear and hierarchical structures compared to the original t-SNE and UMAP based on ED, as well as the PHATE based on the potential distance (PD), in terms of both synthetic and real data sets.

## Methods

### Overview of t-SNE

The t-SNE [1, 2] is a dimensionality reduction method that takes as input a HD data set *X* and returns the LD (usually 2D) coordinates *Y* for the purpose of visualisation of data patterns and organisations. The basic idea of the method is to transform the distances between data points in both of the HD and LD spaces into probability distributions. How well the distances are preserved is then quantified in terms of a dissimilarity measure (or cost function), with the Kullback-Liebler divergence commonly used, between the two distributions. Variants of t-SNE [16–18] differ from each other in the probability distributions and the dissimilarity measure used in the methods.

The t-SNE directly takes as inputs the distances between points without the need to know the coordinates of the HD feature space. It proceeds by first converting the HD distances into a probability distribution $p_{ij}$, usually defined by a Gaussian kernel, over all pairs of points $x_i$ and $x_j$, such that close points have high probability. A key parameter to be set in t-SNE is the ‘perplexity’ which corresponds to the effective number of neighbors covered by the Gaussian kernel (See Additional file 1: Appendix for details). The perplexity therefore controls the variable widths of the Gaussian kernel (or the neighborhood ranges) around different data points in the HD space such that points separated beyond this range are considered to be faraway.

Another key idea of t-SNE is the use of long-tailed t-distribution for the probability distribution $q_{ij}$ associated with $y_i$ and $y_j$ in the LD space. As a result of the mismatching of the two distributions $p_{ij}$ and $q_{ij}$ at large distances, faraway points beyond the neighborhood ranges set by the perplexity in the HD space tend to map to much larger distances in the LD space. This mismatch is claimed to mitigate the crowding problem in dimensionality reduction [1]. Moreover, points within the neighborhood ranges set by the perplexity in the HD space tend to map to points also close in the LD space. These together amplify and better reveal discrete cluster structures provided that an appropriate value of perplexity is chosen. In practice, a default perplexity value of around 30 is often used with the hope that it defines reasonable neighborhood ranges that match with the spatial extents of clusters in the data.

On the other hand, the Kullback-Leibler divergence, given by $\sum _{ij}p_{ij}\log \frac{p_{ij}}{q_{ij}}$, as the cost function is asymmetric in $p_{ij}$ and $q_{ij}$. This means that short distances in the HD space with large $p_{ij}$ contribute significantly to the cost function, whereas long distances with small $p_{ij}$ contribute less. Consequently, this asymmetric property of the cost function tends to prevent close points in the HD space from getting separated in the LD space (i.e., extrusions are discouraged). However, it does not prevent distant points in the HD space from being mapped close in the LD space despite the mismatching of $p_{ij}$ and $q_{ij}$ at large distances mentioned above (i.e., intrusions can occur). The optimisation of t-SNE to find the configuration of points *Y* that minimizes the cost function are generally performed using gradient-based methods. The mathematical details of t-SNE and its optimisation procedures are given in the Additional file 1: Appendix.

### Graph distance motivation

The t-SNE schemes [1, 4] are commonly employed to embed HD data based on, for example, the Euclidean distance (ED) in the HD space. However, many conventional distance measures in the HD space, such as the ED, Hamming distance for data string comparison [19], negative binomial distance for comparison of gene count vectors in single-cell RNA sequencing research [17], etc., are often good distance measures only in local neighborhoods that are small compared to the extents of nonlinear structures in the data. For instance, the ED can only be used locally for data points lying on a hemi-sphere since it fails to capture the curved shape of the underlying manifold when comparing remote points. In other words, conventional distance measures fail to capture the global shape and organisation of the data structures. This poses a problem when the common perplexity value of 30, which can connect moderately remote points, is used to produce LD embedding of distinct clusters, e.g., for the MNIST data set [1]. On the other hand, a choice of small perplexity that focuses only on preserving small local structures could result in a LD embedding composed of many small spurious clusters that do not exist in the HD data [20]. Furthermore, global structure and hierarchical organisation of clusters are likely lost when a small perplexity is used [4, 20].

It is therefore generally difficult to choose an appropriate perplexity that is small enough for conventional distance measures to be useful, but large enough to be able to capture global structures in the HD data. Here we propose to employ the graph distances of the HD data as inputs to t-SNE to resolve the above shortcomings. Graph distances, sometimes called shape-aware distances, better capture the global nonlinear structures where the HD data reside. As will be shown later in the “Results” section, this naturally leads to a choice of large perplexity value that cannot only mitigate the problem with spurious clusters, but also largely preserve the global and hierarchical structures of the HD data.

### Graph construction

The first step in evaluating the graph distances is to construct a graph to represent the HD data, where a node in the graph corresponds to a data point and edges represent the local relationships between points. We define local neighborhoods by only connecting each data point $x_i$ to its *k* nearest neighbors (kNN) based on, e.g., the ED. A graph similarity matrix $w_{ij}$ with $i,j=1,\ldots ,n$ between data points $x_i$ and $x_j$ is defined as the inverse of the squared ED, $1/ \Vert x_i-x_j \Vert ^2$. Some studies also use the Gaussian kernel for $w_{ij}$ [1, 13, 21]. The inverse of squared ED is suggested in this study to avoid introducing the Gaussian width as an additional parameter. The similarities of disconnected data points are simply set to zero. With the similarity matrix $w_{ij}$, the constructed graph can also be viewed as a Markov network with transition probability $w_{ij}/\sum _k w_{ik}$ for a transition from node *i* to node *j*.

Different from the perplexity, the parameter *k* in the graph construction specifies the extent of the local neighourhoods where conventional distance measures, e.g., ED, can be used. We therefore choose a value for *k* that is as small as possible, just to keep the graph connected, that is, for each point $x_i$ one can reach any other data point $x_j$ using only the local connections. Commonly *k* is found to be around 5 with this method. If the data consists of highly disconnected regions, *k* may end up being very large to maintain connectivity in the graph. Nevertheless, this case can be handled by first finding the *k* just large enough for the graph to be connected, then identifying the disconnected components for the ($k-1$)NN graph, which can be done efficiently in linear time by a depth-first search [22]. By keeping the links between the components from the *k*NN graph, one can then re-run the above algorithm recursively on the disconnected components until a lower value of *k*, e.g. around 5, is reached.

### Biharmonic distance

Various graph distances, such as the geodesic distance [23], commute time distance (CTD) [12], diffusion distance [21], etc., exist in defining relationships between nodes that capture the intrinsic geometry of the data. In this study, we employ the biharmonic distance (BHD) [12] to measure distances between points.

Several advantages of employing the BHD are as follows: (i) The BHD between points from the same clusters are usually very small due to the strong within-cluster connectivity in the graph, whereas the BHD between points from different clusters could be very large due to the weak connectivity between clusters. This property of BHD makes discrete structure exaggerated and easier to detect. (ii) Compared with the geodesic distance, the BHD is robust to random noise [12]. (iii) The BHD can be expressed and computed in terms of the eigenvalues and eigenvectors of the graph Laplacian, one of the most fundamental concepts in graph theory [24]. (iv) Unlike, e.g., the diffusion distance, the BHD involves no additional parameter and therefore reduces the subjective input from users. (v) The CTD is similar to the BHD in its computation and points (i)-(iv) holds for the CTD as well. However, a different weighting of the eigenvalues when computing the BHD compared to the CTD leads to a higher stability in estimating large distances [12].

### Validation of the low-dimensional embedding

In order to monitor the preservation of cluster and hierarchical structures by SASNE, t-SNE, PHATE and UMAP, we advocate the use of quantitative validation indices to compare and evaluate the quality of the LD embedding. In previous studies [1, 2], quality of the embedding are often carried out by simple visual inspections but this may lead to misleading conclusions about the data by interpreting spurious patterns created by the methods. To provide a quantitative account of the merits of SASNE compared with the competing methods at the point-wise, cluster-wise (or intermediate), and inter-cluster (or global) scales, we introduce two complementary validation indices, one for clustering and another for dimensionality reduction as follows.

### Cluster validation

In this study, we evaluate how faithfully the embedding preserves the underlying clusters using the silhouette index [25]. For a given point $x_i$ assigned to the cluster $C_k$ ($k = 1,\ldots , K$ with *K* the number of clusters) containing $N_k$ points, the cohesion $a_i$ is defined as $a_i = \frac{1}{N_k} \sum _{j :j \in C_k} \delta _{ij}$ where $\delta _{ij}$ denotes the distances between points $x_i$ and $x_j$ and the sum runs over all points in the same cluster $C_k$. Here $\delta _{ij}$ is the conventional distance measure, e.g. ED, when the t-SNE or UMAP are used, the BHD when the SASNE is used and the PD when PHATE is used.

To quantify separation, we first define a point-to-cluster distance $\delta (x_i,C_l) = \frac{1}{N_l}\sum _{j:j \in C_l} \delta _{ij}$ where the sum runs over all points in the cluster $C_l$. For a given point $x_i$ in the cluster $C_k$, the separation $b_i$ is defined as the distances from $x_i$ to the closest cluster that $x_i$ does not belong to, i.e., $b_i = \min _{l \ne k} \delta (x_i,C_l)$. Combining the cohesion and separation, the point-wise *silhouette value* $s_i$ for point $x_i$ can then be defined as

1 $$\begin{aligned} s_i = \frac{b_i - a_i}{\max (a_i,b_i)}. \end{aligned}$$

One can see that $-1\le s_i\le 1$ and $s_i$ is close to 1 ($-1$) for a good (bad) clustering with large (small) separation $b_i$ and small (large) cohesion $a_i$. Furthermore, the cluster-wise silhouette score $\overline{s}_k$ can be naturally evaluated as the average silhouette value over all points in the cluster $C_k$,

2 $$\begin{aligned} \overline{s}_k = \frac{1}{N_k} \sum _{i : x_i \in C_k} s_i \end{aligned}$$

Finally, an overall silhouette coefficient $\overline{S}$ is evaluated by averaging over all clusters,

3 $$\begin{aligned} \overline{S} = \frac{1}{K} \sum _{k = 1}^K \overline{s}_k. \end{aligned}$$

We first note that the silhouette index is primarily designed to validate clustering (i.e., unsupervised learning) methods in which the data do not come with labels. Nevertheless, we will apply the silhouette index in “Results” section below to our test and real data sets whose clusters $C_k$ are known, to evaluate how well clustering structures are preserved from the HD space to the LD embedding.

To correctly evaluate clustering results with non-spherical clusters, conventional distance measures, e.g., the ED, which does not contain any shape information, should not be used as the distances $\delta _{ij}$ in the silhouette index. Instead, we will show in “Results” section that the use of the BHD is more appropriate. On the other hand, the separation $b_i$ in the silhouette index only considers the closest cluster to the data point under consideration. This means that the silhouette index cannot validate how well hierarchical organisations of clusters at the inter-cluster scales are preserved by the LD embedding. This leads us to introduce a complement validation method that takes the relative placement of the data points into account.

### Dimensionality reduction validation

We complement the silhouette index by quantifying how well the relative placement of points in the LD space agrees with those in the HD space. In dimensionality reduction, preservation of exact distances is too restrictive that can seriously hamper the flexibility of the nonlinear mapping from the HD to the LD space [26]. Instead, it is more desirable for the embedding to only impose a monotonic relationship between the HD and LD distances that corresponds to the preservation of distance rank ordering [27–29]. Unlike classical methods such as PCA and multidimensional scaling, t-SNE and UMAP do not aim at preserving exact distances.

In this study, a rank-based validation scheme for dimensionality reduction is formulated as follows. For each point $x_i$ in the HD space, the rank vector $\varvec{r_i}^{x} = (r^{x}_{ij})_{j \ne i}$ is defined, where $r_{ij}^{x} = r$ if $x_j$ is the *r*th closest point to $x_i$. The rank vector $\varvec{r}_i^{y}$ is defined in the same way for the LD space. We then define a point-wise quality measure, $\overline{\varvec{r}}_i$, for the point $x_i$ as the mean absolute rank error (MARE),

4 $$\begin{aligned} \overline{\varvec{r}}_i = \frac{1}{n-1} \sum _{j:j \ne i} \frac{\mid r^{x}_{ij} - r^{y}_{ij} \mid }{n-1} , \end{aligned}$$

to quantify how well the embedding from the HD to LD space preserves the distance ordering relative to the point $x_i$. Here the MARE is normalised to lie between 0 (perfect rank preservation) and 1 (complete distortion of ranks). Likewise, an overall quality measure of preservation of rank ordering that we term ‘average rank error’, $\overline{R}$, can be evaluated by averaging the point-wise quality over all data points,

5 $$\begin{aligned} \overline{R} = \frac{1}{n}\sum _{i = 1}^n \overline{\varvec{r}}_i. \end{aligned}$$

In addition to the quality measures, it is informative to create the rank residual plot (RRP) that allows us to visually inspect the distribution of the rank residuals $r^{x}_{ij} - r^{y}_{ij}$. The RRP is a 2D density plot whose ordinate and abscissa are the value of the normalised rank residuals $(r^{x}_{ij} - r^{y}_{ij})/(n-1)$ and the normalised original rank index $j/(n-1)$ ($j=1,\ldots ,n-1$), respectively. As we will see in the “Results” section, the RRP also tells us at what scale and to what degree the distance orderings are distorted in the embedding.

## Results

### Simulation studies

To demonstrate the advantages and provide insights for our graph-based approach, we apply the t-SNE, PHATE, UMAP and the SASNE to four simulated test data sets whose clustering structures are known beforehand. These test sets aim to represent different types of data with features that are often found in real data, allowing us to highlight the merits of using graph distances in cluster separation, dimensionality reduction quality and visual clarity.

**Fig. 1:**
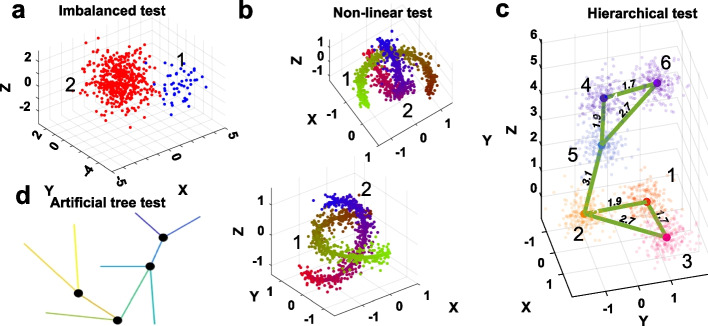
Four synthetic data sets. **a** Data sampled from two Gaussians with equal covariance matrix but different means. The red and blue clusters contains 1000 and 50 points, respectively. **b** Data sampled uniformly along two non-overlapping 1D nonlinear curves with Gaussian noise added. Each cluster contains 400 points. **c** Data contains 6 clusters with 100 points each. Data are sampled from Gaussians with equal covariance. The cluster means are arranged in two major groups, each containing 3 sub-clusters. The green lines are included for clarity. The numbers next to the lines indicate the ED between the cluster means. **d** Schematic illustration of data set containing 1440 points sampled on piece-wise linear manifolds in 60 dimensional space with Gaussian noise added to all the 60 dimensions

We show in Fig. 1a the first test case of ‘imbalanced clusters’. Two clusters are generated from 3D Gaussian distributions with the same variance but with different means and number of points. A good LD (2D) embedding is expected to clearly separate the two clusters.

The second test case where clusters have ‘nonlinear structure’ is shown in Fig. 1b. Each cluster contains 400 points that are sampled along the two underlying 1D curves with Gaussian noise added. A good LD embedding is expected to not only reveal the 1D underlying structures, but also place the data correctly into two distinct groups.

The third test case shown in Fig. 1c simulates a data set with ‘hierarchical structure’, where clusters 1 to 3 and clusters 4 to 6 form two distinct ‘super-clusters’, respectively. A good LD embedding is expected to reveal this hierarchical structure where the rank ordering of the distances between the six cluster centers is preserved.

We show silhouette plots in Additional file 2: Fig. S1 for the three test cases above comparing the BHD, ED and PD before any embedding. It is informative to see the advantage of using the BHD over ED and PD in highlighting clustering structures.

The fourth test case, also studied by Moon et al. [13], is illustrated in Fig. 1d. This data set contains no discrete structure. Instead, the data mimics continuous developmental trajectories that branch off in various directions. This structure is common in single-cell data, for example, where cell types continuously differentiate into other kinds of cells. A good LD embedding should therefore reveal the different developmental branches and correctly maintain their continuous structures.

### Choice of hyper-parameters

The usual rationale in choosing low perplexity around 30 in the t-SNE is to preserve the local neighborhoods as well as possible. It was also claimed that the t-SNE results are fairly robust against a change of perplexity value [1]. However in terms of distance rank preservation, a low perplexity is in fact a poor choice, whereas choosing large perplexity value, such that the Gaussian kernel can cover remote points, consistently results in significant improvements (see Additional file 2: Fig. S2), especially for the SASNE. We therefore propose a natural choice of perplexity to be around $90\%$ of the number of points for SASNE, and use this large perplexity value in all the following analyses as a default value. Choosing $90\%$ also allows us to exclude outliers that can result in extremely wide Gaussian kernels. We note that perplexity equal to the number of points is not possible since this would correspond to an infinite bandwidth of the Gaussian kernel with all neighbors weighted equally (see Additional file 1: Appendix).

As apparent cluster structures frequently appear in the LD embedding, it may be tempting to choose a lower perplexity when assessing the performance of t-SNE or SASNE qualitatively by eye. However, due to the possible appearance of spurious clusters and the loss of relative placement of clusters, we do not suggest for such choice to avoid making misleading conclusions about the data. For t-SNE, UMAP and PHATE, we follow the default hyper-parameter settings proposed in the original works (see Additional file 1: Appendix).

### Dimensionality reduction validation

**Fig. 2:**
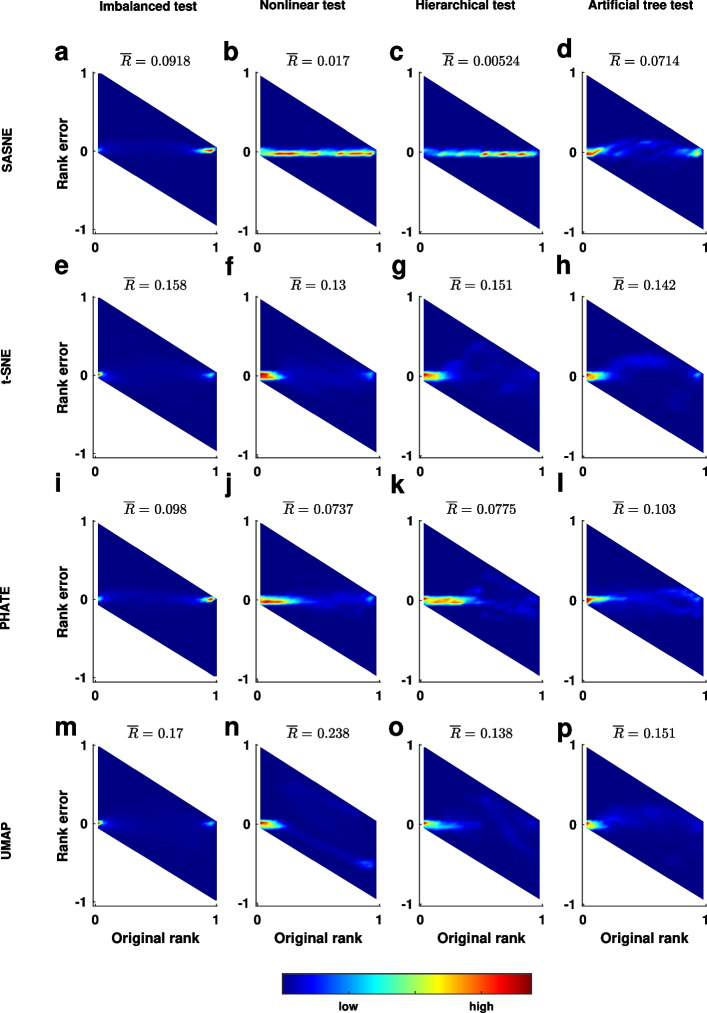
Rank residual plots (RRP) for the four simulated test cases. The perfect situation in which all distance rank orderings are preserved in the embedding implies that all residuals equal to zero. In that case, the RRP shows a shape peak along the horizontal line in the middle of the plot. The residuals are visualised via a 2D histogram, where each bin is colored according to the relative density of points, according to the color bar at the bottom of the plot. Unreachable regions are colored white. The values on the top of each RRP correspond to the average rank error $\overline{R}$. The test cases are arranged per column with the same order as in Fig. 1

Figure 2 shows the RRPs and the average rank errors, $\overline{R}$, for the four test cases embedded by the methods. The RRPs show the distance rank preservation at all scales. In particular, distortion of small (large) ranks corresponds to error on the local (global) scale. The local and global scales locate on the left and right sides in the RRP, respectively.

In case of the imbalanced data set (first column in Fig. 2), many rank orderings, especially at the intermediate scales located in the middle portion of the abscissa in the RRP, are not accurately preserved. This is expected since three variables are required to describe the relationship between the points from a 3D Gaussian distribution. Nevertheless, the SASNE and PHATE show high rank preservation at the large (inter-cluster level) and small scales are comparable in this test case with simple spherical cluster structures. In contrast, UMAP and t-SNE show poor rank preservation for the larger distances with slightly better preservation for the local neighborhoods. They have a high average rank error compared to SASNE and PHATE.

From the second column of Fig. 2, we observe for the nonlinear case a significant improvement of the distance rank preservation in the SASNE compared to all other methods, especially t-SNE and UMAP. The RRP shows that the rank ordering at all scales are highly preserved in SASNE (Fig. 2b). This is a direct consequence of using the shape-aware distance, BHD, that is able to capture the underlying nonlinear structure where the data points reside on.

The RRPs of the embeddings of the hierarchical data set by t-SNE, PHATE and UMAP (Fig. 2g, k, o) show that mainly the small ranks are preserved while the large ranks are distorted to a higher degree. This means that the hierarchical organisation of the clusters is lost in these embeddings. On the other hand, the high preservation of distance ranks by SASNE is shown in Fig. 2c. The main improvement is from the preservation of the large distance ranks, meaning that the hierarchical organisations of the clusters are well preserved in the embedding. This also results in a significantly lower average rank error compared to the other methods.

The RRPs evaluating the embeddings of the artificial tree test are shown in the fourth column of Fig. 2. The t-SNE and UMAP preserve mainly the small distance ranks with poorer preservation of the large distance ranks compared to SASNE and PHATE, which results in the higher average rank errors. The SASNE achieves the lowest average rank error with slightly better preservation of the large distance ranks compared to PHATE.

### Evaluation of the LD embeddings

**Fig. 3:**
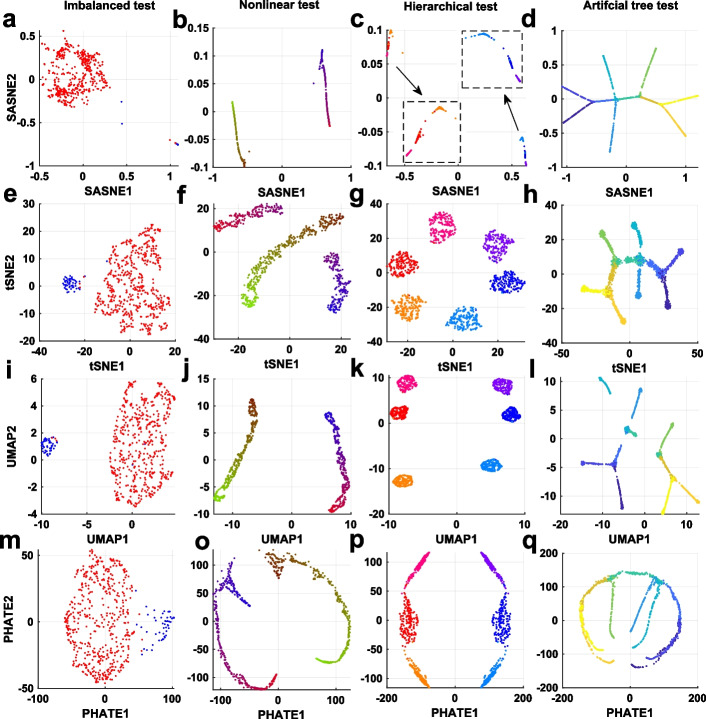
2D embeddings of the test cases in Fig. 1. The color scheme of the clusters are the same as in Fig. 1. The insets in **c** magnify the cluster structures separated far apart from the embedding

**Fig. 4:**
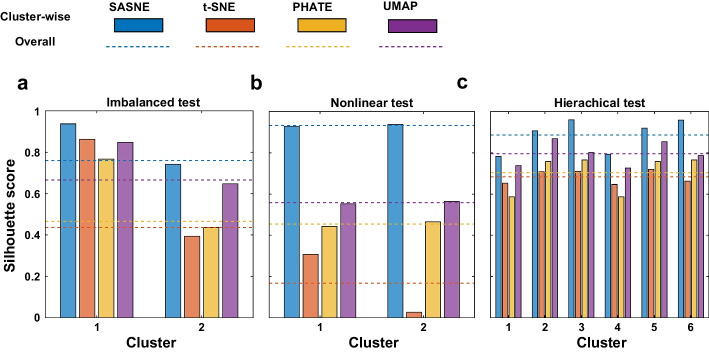
Barplot showing the average silhouette value for each of the simulated test cases, where clusters are present, and for each method. The dashed lines corresponds to the silhouette coefficient. **a** Barplot showing average silhouette value together with silhouette coefficient for each method for the imbalanced test. **b** Barplot showing average silhouette value together with silhouette coefficient for each method for the nonlinear test. **c** Barplot showing average silhouette value together with silhouette coefficient for each method for the hierarchical test

The RRPs in Fig. 2 show that the preservation of the distance rank is best for SASNE compared to other methods in all test cases. Next we gain more insights on this superior performance by looking at the corresponding LD embeddings. The resulting LD embeddings of the imbalanced data is shown in the first column of Fig. 3. The SASNE gives very distinct cluster separation that clearly reveals the discrete structure. Indeed, the silhouette coefficient and average silhouette value shown in Fig. 4a for the embedding confirm the superior ability of SASNE in highlighting clusters. Furthermore, the cluster separation in the UMAP plot is comparable to SASNE. The t-SNE and PHATE demonstrate less clear separation of the clusters, where it could be difficult to visually identify the clusters and distinguish it from spurious patterns created by the algorithm.

For the nonlinear data set shown in the second column of Fig. 3, one can see one limitation of the t-SNE that it fragments one of the two clusters into two spurious clusters. This results in a low silhouette score and average silhouette value shown in Fig. 4b. The SASNE, UMAP and PHATE successfully untangle the two shapes. Furthermore, the SASNE (Fig. 3b) achieves better denoising of the data, thereby clearly revealing the underlying 1D structures of the clusters. This improvement in the clustering quality is further confirmed by the silhouette coefficient in Fig. 4b.

The embeddings of the hierarchical data set are shown in the third column of Fig. 3. At first glance, the t-SNE and UMAP may be preferred as the spherical shapes of the clusters from the original 3D data (Fig. 1c) are retained. However, the RRPs in Fig. 2g, o show that both t-SNE and UMAP introduces distortions in distance ranks at most scales except for the very local scale, implying their failure in retaining the global arrangements of the clusters. On the other hand, the SASNE achieves much lower distance rank distortion at all scales as shown in the third column of Fig. 2b, implying that the hierarchical structure of the clusters is well preserved. In terms of cluster validation, the t-SNE correctly separates the individual clusters within each group. The method is however not able to clearly distinguish the reddish group (clusters 1–3) from the bluish group (clusters 4–6) as shown in Fig. 3g. The UMAP is also not able to preserve well the distance ranks of cluster separations when comparing to Fig. 1c. Similarly, PHATE depicts the two groups symmetrically that does not reflect the true structure. A better separation of the clusters within each group is obtained by the SASNE, confirmed by the silhouette coefficient shown in Fig. 4c.

The embeddings of the artificial tree data is found in the fourth column of Fig. 3. The SASNE embedding clearly shows the different branches of the tree while also keeping the trajectories intact. Furthermore SASNE denoises the data and clearly shows the 1D structure of the trajectory. Despite having less denoising compared to SASNE, the t-SNE also performs well on this data set by keeping the tree connected. Crucially, UMAP shatters the tree and produces several spurious clusters. Although PHATE is able to keep the tree connected, some branches are merged together, meaning that the relative positioning of the branches is lost from the embedding.

In summary, the above test cases demonstrate that the SASNE can reliably embed and reveal clusters with imbalanced, arbitrarily shaped and hierarchical structures based on the qualities of both clustering and preservation of distance ranks. It also prevents creating spurious discrete structures that shatter continuous trajectories in the data. Since the shape-aware BHD provides us with a valid global distance measure, the choice of a larger perplexity value, e.g., 90$\%$ of the number of points, allows us to consistently fix the only hyper-parameter of the embedding method in a data-driven way. To demonstrate the superior performance of SASNE for real HD data, we consider the following two data sets.

### Gene expression data

We consider a data set of gene expressions from 3663 cells taken from the hippocampal area of a mouse brain [17]. Each cell is characterised by a gene count vector, indicating the expression frequency of the sequenced genes. With the gene count vector as coordinates of the HD space, the data set allows us to identify groupings of cells that correspond to distinct cell types based on their gene expression profiles. In contrast to the simulated data sets and the MNIST data discussed in the next section, the gene expression data is unlabelled, i.e., the corresponding clusters, or cell types, to which the cells belong to are unknown beforehand. Therefore, an additional clustering procedure (not performed here) is needed to group the data points in the LD embedding. Since no cluster label is available, we focus only on how well distance ranks are preserved in the LD embedding and do not consider cluster validation in this case.

**Fig. 5:**
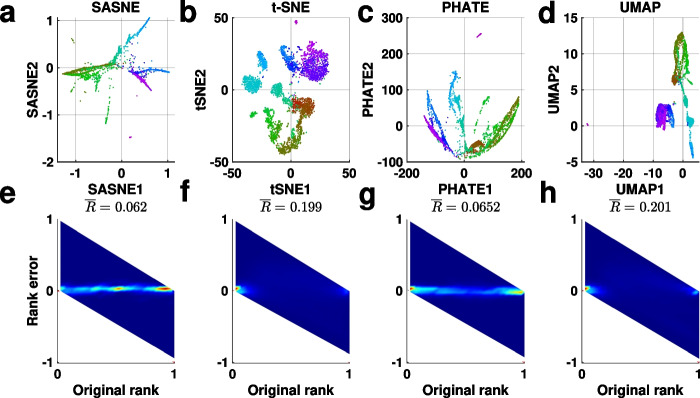
Results of applying SASNE, t-SNE, PHATE and UMAP to the single-cell data set. **a**–**d** Resulting LD projections by SASNE, t-SNE, PHATE and UMAP, respectively. Each data point is colored according to the clustering results of Harris et al. [17]. **e-h** RRPs for each of the LD embeddings

Before applying the methods, we follow the same procedures performed by Kobak et al. [4] to reduce the number of features that produce comparable results to those reported by the original works [17] where the data set was obtained. Specifically, we select 1000 representative genes out of 27998 in total that show high expression levels in a smaller subset of cells, indicating their capability of being good molecular features to distinguish cell types (see Additional file 1: Appendix). The resulting embeddings of the gene expression data are shown in Fig. 5a–d. For comparison, the data is colored according to a previous clustering result performed by Harris et al. [17] that gave rise to a total of 49 clusters by fitting a mixture of binomial distributions using the expectation maximisation algorithm. It has been reported that these cell clusters form hierarchies, where clusters close to each other are indicated by similar colors, often with continuous transitions between clusters. Therefore, a better preservation of distance ranks in the LD embedding is important to correctly embed these hierarchies and to preserve continuous developmental trajectories in order to provide meaningful biological interpretations.

From Fig. 5a, the SASNE corroborates the previous clustering result that cell groups colored similarly also fall into nearby regions in the SASNE space. For the preservation of hierarchical structures, the RRP shown in Fig. 5e confirms a relatively low degree of rank distortion across all scales. Moreover, SASNE shows a pronounced improvement compared to the t-SNE and UMAP according to the RRPs and average rank errors shown in Fig. 5f, h. Although clustering validation was not performed for this data set, one can still see from Fig. 5b, d that the t-SNE and UMAP displays better discrete data structures, but with a large distortion of distance ranks across all scales, likely shattering continuous transitions between clusters. Moreover, the PHATE achieves a comparable average rank error, but with a higher distortion of the intermediate distance ranks, as can be seen in Fig. 5e, g. By examining the PHATE embedding in Fig. 5c, we note the similar shape compared to the artificial tree embedding in Fig. 3q, i.e., some trajectories appear to be merged by the PHATE. On the other hand, the SASNE shows more distinct developmental trajectories in the LD embedding indicated by Fig. 5a, c, while simultaneously achieving slightly higher preservation of distance ranks as seen by comparing Fig. 5e and **g**.

### MNIST handwritten digit data set

We now apply the methods to the MNIST data set consisting of gray scale images of handwritten digits [30]. Each image is represented by a 784 ($28 \times 28$) dimensional vector whose entries correspond to the pixels of the image. The images are labelled based on which digit, from 0 to 9, it corresponds to. This enables us to evaluate how well the images are grouped according to their labels in the LD embedding without the need for extra clustering procedures.

The MNIST data set has known hierarchical structures. For example, digits 4 and 9 look more alike to each other compared to digits 4 and 1. This is confirmed by examining the overlaps between the digits (see Additional file 2: Fig. S3). Moreover, all digits overlap with the other digits to some extent, with only digits 0, 1 and 6 showing relatively clear separations from the other digits. The continuous overlapping between images from different digits should be reflected in the LD embedding. Moreover, some digit clusters are non-spherically shaped (see Additional file 2: Fig. S4) indicating the advantage of using shape-aware distance measures.

By examining the silhouette plots of different distance measures in the original HD space (see Additional file 2: Fig. S5), one sees a low silhouette coefficient for all distance measures, again indicating significant overlap between the digits. In particular, the silhouette plots for BHD indicate that digits 0, 1, and 6 have relatively large (digit-wise) silhouette values, consistent with the overlapping analysis in Additional file 2: Fig. S3 showing that these digits are the most distinct. The remaining digits do not show clear separation according to the silhouette plot, consistent again with the overlapping analysis (Additional file 2: Fig. S3). On the other hand, the ED gives a silhouette coefficient close to 0, indicating that the digits do not form discrete clusters according to the ED. The PD has a comparable silhouette coefficient to the BHD (Additional file 2: Fig. S5) that confirms the advantage of using graph distances when handling clusters with arbitrary shapes. Nevertheless, the silhouette plots of PD indicate that all digits separate roughly equally in the HD space. For example, digit 1 and 2 have similar silhouette values, inconsistent with the overlapping analysis (Additional file 2: Fig. S3).

**Fig. 6:**
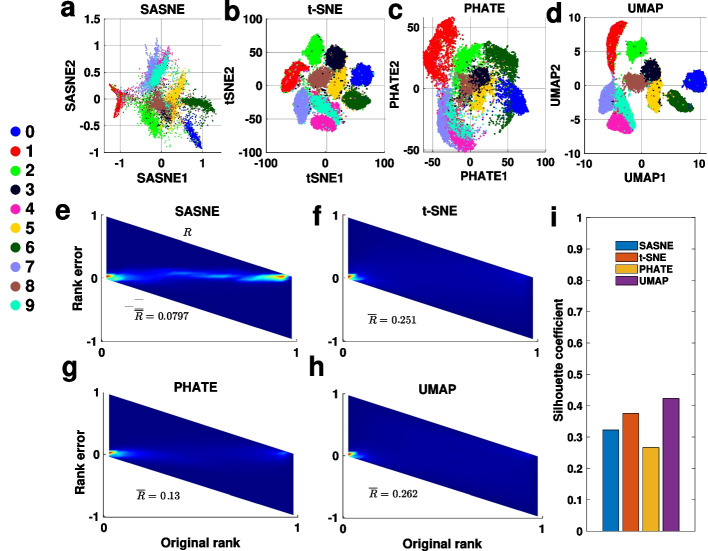
Results of LD embeddings by SASNE, t-SNE, PHATE and UMAP. **a**–**d** 2D projections of the MNIST data set using SASNE, t-SNE, PHATE and UMAP respectively. Each point is colored according to what digit it represents. **e**–**h** RRPs for the LD embeddings by SASNE, t-SNE, PHATE and UMAP respectively. **i** Barplot showing the silhouette coefficient evaluated on the LD embedding on the MNIST data set for each method

The resulting 2D SASNE with an appropriate perplexity chosen to be $90\%$ of the number of data points (see Additional file 2: Fig. S7) is shown in Fig. 6a. The embedding shows that digits 0, 1 and 6 form relatively distinct clusters, whereas, e.g., digit 2 overlaps with digits 1, 3, 7 and 9, which is consistent with the overlapping analysis (Additional file 2: Fig. S3). Indeed, the RRPs in Fig. 6e–h show the significant improvement by SASNE in preservation of the relative placement of the clusters in the LD embedding by SASNE compared to the other methods. On the other hand, although the UMAP embedding shows clearly separated clusters (Fig. 6d), it fails to capture the overlaps between digits. This can be seen by both the UMAP and t-SNE embedding (Fig. 6b, d) of the digit 2 that is incorrectly separated from the digit 7. The PHATE captures the overlap between digits more accurately compared to UMAP and t-SNE. Nevertheless, digits that are relatively well separated are not reflected in the PHATE embedding. For example in Fig. 6c, digit 0 and 6 are merged, and digit 1 is not separated from digit 2 and digit 7, which are inconsistent with the overlapping analysis (Additional file 2: Fig. S3).

In terms of the clustering quality, all methods result in a relative low overall silhouette coefficient compared to the test case (see Fig. 6i). This is expected due to the small separations, $b_i$, in the point-wise silhouette value in Eq. 1. The slightly higher silhouette coefficients of the UMAP and t-SNE indicate that discrete structures are more profound in the embedding (Fig. 6b, d). However, the profound discrete structures appeared in these embedding are likely to be spurious that do not reflect the true data structures revealed by the overlapping analysis (Additional file 2: Fig. S3) and the grouping of the digits in the original HD space (Additional file 2: Fig. S5). The PHATE has the lowest cluster separation and does not clearly reveal the distinct separation of digits 0, 1 and 6. In contrast, for digits 0, 1 and 6 the digit-wise silhouette scores are higher in the SASNE than in all other methods (see Additional file 2: Fig. S6). These all together demonstrate the ability of the SASNE to amplify true discrete structures in the data and preserve the relative organisations among these discrete patterns.

To sum up the analyses of the MNIST data set, the SASNE performs well simultaneously in clustering quality and preservation of distance ranks and hierarchical structure. Although UMAP and t-SNE show discrete structures, this is often not an accurate representation of the HD data, where overlaps and hierarchical structure may be lost in the LD embedding. The PHATE shows better preservation of distance ranks compared to t-SNE and UMAP, but is relatively weak in revealing discrete structures in the data.

## Discussion and conclusions

By incorporating the concept of shape-aware distances we have in this study proposed the SASNE. We have shown how it can mitigate some of the shortcomings of the t-SNE, UMAP and PHATE methods. This is done in a data-driven way that can consistently fix the hyper-parameter, perplexity, of the method. In terms of quantitative validation methods in both clustering and dimensionality reduction, the advantages of SASNE were exemplified with synthetic imbalanced, nonlinear, hierarchically structured data, and developmental trajectories where the ground-truth is known. The methods were then applied to two real HD data sets; the single-cell gene expression data and the MNIST handwritten digits data set. In both the synthetic and real data sets, the SASNE demonstrates superior performance compared with the current state-of-the-art methods t-SNE, UMAP and PHATE in capturing discrete and hierarchical structures hidden in the HD feature spaces.

It has been claimed in certain cases that the UMAP can outperform t-SNE in computational speed and preservation of global structures [14, 15]. Nevertheless, it was found [4] that the performance of the two methods depends highly on the hyper-parameter settings, and their results could be similar for certain choices of hyper-parameters. Moreover, distortion of large distance ranks by both t-SNE and UMAP is expected, as both methods do not attempt to preserve global distances. The PHATE method is similar to SASNE in that it aims at preserving the graph distance, PD, that can correctly estimate long range distances. As our experiments show, however, the PHATE embedding does not accurately reveal the discrete structures to the same extent as the SASNE. We identify two potential reasons for this. First, the PD is defined as the logarithmic transformed diffusion distance that introduces a hyper-parameter *t* controlling the time-scale of the diffusion. A single time-scale *t* often cannot capture both local and global scales and therefore multiple values of *t* should be examined to get a complete picture [12, 21]. Second, PHATE relies on the metric multidimensional scaling (MDS) to embed the PD into the LD space. However, the MDS aiming to preserve all distances as much as possible has been shown to perform poorly compared to t-SNE due to its inability to handle the crowding problem in the LD embedding [1].

Some other related studies making use of graph-based methods to improve the performance of t-SNE also exist. In particular, Parviainen et al. proposed the Graph-SNE (GSNE) [31] that considers the probability for a random walker to reach data point *i* from point *j* and vice versa in a fixed time $\tau$. This probability was then used as the HD distribution $p_{ij}$ in the t-SNE procedures. GSNE has the advantage that it speeds up the evaluation of $p_{ij}$ without the need to perform matrix diagonalisation as in the SASNE. Similar to the PHATE, there is no good strategy in choosing the hyper-parameter $\tau$ that is crucial in determining the ‘scale’ of the regions explored by the random walker in the graph. Therefore, it was suggested [31] to examine a wide range of diffusion times $\tau$ when using GSNE to capture hierarchical structures in the data, which in turn requires several runs of the t-SNE optimisations with increasing computational cost. Another variant of t-SNE is the Hierarchical-SNE (HSNE) [32]. The method speeds up the computations by a landmarking strategy where transition probabilities $p_{ij}$ are approximated by Monte Carlo estimation of simulated random walks on the graph representation of the data. Although there are computational benefits to the approach, there are a multitude of hyper-parameters that need to be determined for the graph construction and estimation of $p_{ij}$.

We finally note that the computational cost of SASNE may become demanding when the data size *n* becomes large, which is an important direction for future improvement. The main computational bottlenecks of SASNE are (i) computation of the BHD matrix and (ii) the gradient based optimisation method used in the original t-SNE implementation which creates the LD embedding from the BHD matrix. Both (i) and (ii) are in the order of $O(n^2)$. As for (i), one approach to reduce the time complexity is to coarse grain the weighted graph [33]. Regarding (ii), numerical approximations have already been proposed to speed up the t-SNE optimisation in which the computational time can be reduced to $O(n\log n)$ by tree-based methods [2], and even to *O*(*n*) by fast Fourier transform and polynomial interpolation [34]. These approximations do, however, rely on the use of low perplexity values that would sacrifice preservation of global structure. Instead we suggest using a stochastic gradient descent method such as Adam [35] to speed up the optimisation.

## Supplementary Information

**Additional file 1**. PDF file containing the Appendix.

**Additional file 2**. PDF file containing supporting figures S1–S7.

## Data Availability

The codes and test data sets are available at https://github.com/tobiaswangberg/SASNE.git.
