# Supplementary material for: Shape-aware stochastic neighbor embedding for robust data visualisations
Source: BMC Bioinformatics. 2022 Nov 14;23:477. doi: 10.1186/s12859-022-05028-8 (PMC9660178; doi:10.1186/s12859-022-05028-8)
Supplement: Supplementary file 1 — Additional file 1. PDF file containing the Appendix. [file 12859_2022_5028_MOESM1_ESM.pdf]

Appendix

Formalism of t-SNE Here we provide some mathematical details of the t-SNE method. Suppose there are n data points, the first step is to transform the distances in the HD space into a probability distribution. Specifically, a 'directed' measure of similarity from point x_i to point x_j in the HD space (with $i, j = 1, \ldots, n$) is defined as a conditional probability in terms of the Gaussian kernel and the softmax function,

$$p_{i|j} = \exp\left(-\frac{\delta_{ij}^2}{2\sigma_j^2}\right) \Big/ \sum_{k \neq j} \exp\left(-\frac{\delta_{kj}^2}{2\sigma_j^2}\right), \qquad i \neq j. \tag{1}$$

The self similarity $p_{i|i}$ is set to 0. Here δ_{ij} denotes the distance between points x_i and x_j, which is the conventional distance measure, e.g. ED, in the t-SNE and the BHD in the SASNE. The variable standard deviations σ_j (with $j = 1, \cdots, n$) can be fixed by choosing a constant value for the perplexity, $\mathcal{P}$, defined by

$$\mathcal{P} = 2^{H(p_{\cdot|j})}. \tag{2}$$

In Eq. (2), $H(p_{\cdot|j})$ denotes the Shannon entropy [1] of the probability distribution $p_{\cdot|j}$, defined as $H(p_{\cdot|j}) = -\sum_{i \neq j} p_{i|j} \log p_{i|j}$.

The perplexity can vary between 1 and $n - 1$ and it corresponds to the effective number of neighbors around a point x_j covered by the Gaussian kernel with standard deviation σ_j. Points beyond the perplexity range will simply be counted as 'faraway'. When perplexity equals 1, it corresponds to the case $\sigma_j \to 0$ that all probability mass is placed on the nearest neighbor. On the other hand, when perplexity equals $n-1$, it corresponds to the case $\sigma_j \to \infty$ in which all neighbors are weighted equally. The perplexity is the main hyper-parameter of t-SNE methods that needs to be determined. Moreover, the probability distribution is symmetrised as $p_{ij} = \frac{p_{i|j} + p_{j|i}}{2n}$ for computational convenience.

Similarly, the distances in the LD embedding space are also transformed into a probability distribution in terms of the long-tailed t-distribution with one degree-of-freedom as follows

$$q_{ij} = \frac{(1 + ||y_i - y_j||^2)^{-1}}{\sum_{k \neq l}(1 + ||y_k - y_l||^2)^{-1}}, \qquad i \neq j. \tag{3}$$

Here in the LD embedding space, the ED is used for both the t-SNE and SASNE. The self-similarity q_{ii} is again set to zero.

The LD embedding coordinates y_i are then obtained by minimizing the Kullback-Leibler (KL) divergence, $\mathrm{KL}(p, q) = \sum_i \sum_{j \neq i} p_{ij} \log \frac{p_{ij}}{q_{ij}}$, as a cost function between the probability distributions, p_{ij} and q_{ij}, using gradient-based methods. The KL divergence has the property that $\mathrm{KL}(p, q) = 0$ if and only if $p_{ij} = q_{ij}$ for all i and j.

t-SNE optimisation The optimisation procedures of t-SNE are as follows: In both the t-SNE and SASNE methods, one minimises numerically the KL divergence between the probability distributions, p_{ij} and q_{ij}, as described above by gradient

descent. Since the cost function is not convex, the optimisation may converge to a local minimum and therefore the solution may depend on the initialisation, i.e., the initial configuration of the coordinates y_i with $i = 1, \cdots, n$ in the LD space.

In case of optimising t-SNE, we follow the protocol of Kobak and Berens [2] that the optimisation is initialised with the two leading principal components of the HD data set, normalised by the standard deviation of the corresponding principal component. The initial configuration is further multiplied by a factor of 10^{-4} which was shown empirically to speed up the convergence. For the SASNE optimisation, we use a similar initialisation procedure as in the case of the t-SNE but apply it to the BHD.

We also adopted the optimisation trick to multiply all HD probabilities p_{ij} by a constant $\alpha = 12$, called early exaggeration, for the first 250 iterations. which was shown to lead to better cluster separation [3]. Moreover, as originally suggested by Belkina *et al.* [4], the learning rate in the gradient descent is set to $\eta = n/\alpha$, where n is the number of points, which has shown to lead to improved convergence behaviour in terms of stability and speed. Given the above settings, the optimisation was performed by the `tsne` function provided by the `MATLAB` Statistics and Machine Learning Toolbox.

Hyper-parameters of PHATE and UMAP Default parameters for PHATE are used as suggested in the original publication [5] and for UMAP we follow the default parameters used by Becht *et al.* [6].

Implementation All methods are implemented in `MATLAB` version `2022a`

Computing the biharmonic distance Given a graph G defined by a $n \times n$ similarity matrix W with elements $w_{ij} = 1/\|x_i - x_j\|^2$, one can compute the graph Laplacian $L = D - W$ [7]. Here D is the diagonal degree matrix with elements $d_i = \sum_k w_{ik}$ that is the degree of the node i. The BHD between the points x_i and x_j can be expressed in terms of the eigendecomposition of L as $C_{ij} = \mathrm{Vol}(G) \sum_{k=2}^{n} (v_{ik} - v_{jk})^2/\lambda_k^2$ [7], where λ_k is the kth eigenvalue, v_{ik} is the ith element of the kth eigenvectors of L, and and $\mathrm{Vol}(G) = \sum_i d_i$ is the volume of the graph G. This expression also shows that the BHD has the form of an ED, i.e., sum of squares $\sum_{k=2}^{n} (z_{ik}^2 - z_{jk}^2)$, with the $(n-1)$D Euclidean coordinates for the ith data point given by $z_{ik} = v_{ik}/\sqrt{\mathrm{Vol}(G)/\lambda_k^2}$ $(k = 2, \cdots, n)$. A convenient property of these coordinates are that the corresponding covariance matrix is diagonal. Therefore the PCA initialisation based on the BHD is simply the leading coordinates with largest corresponding eigenvalues, in this Euclidean space. Hence, after computation of the eigendecomposition of L, these coordinates can be directly input to any standard t-SNE implementation where we keep optimisation scheme consistent with the original t-SNE algorithm.

In this study we computed the BHD using the symmetric Laplacian $L^{\mathrm{sym}} = D^{-\frac{1}{2}} L D^{-\frac{1}{2}}$ [7], instead of L, since this resulted empirically in better results based on the RRP. The computation of the graph distance is now based on the modified formula compared to the one above, given by $z'_{ik} = v'_{ik}/\sqrt{\mathrm{Vol}(G)/d_i {\lambda'}_k^2}$. Here $v'_{\cdot k}$ and λ'_k denotes the eigenvector and eigenvalue of L^{sym} respectively. Notice that the distance given by $C'_{ij} = \sum_{k=2}^{n} ({z'}_{ik}^2 - {z'}_{jk}^2)$ is not equal to the original BHD in general.

Pre-processing of single-cell data We follow the same pre-processing procedures as used by Kobak *et al.* [2] as follows: Let n_c and n_g be, respectively, the number of cells and the number of genes under consideration. We denote x_{ig} as the expression level of gene g ($g = 1, \cdots, n_g$) in cell i ($i = 1, \cdots, n_c$). The fraction of cells that do not express the gene g is given by $d_g = \frac{1}{n_c} \sum_{i=1}^{n_c} I(x_{ig} = 0)$, where the indicator function $I(x_{ig} = 0)$ is equal to 1 when $x_{ig} = 0$, and 0 otherwise. Furthermore, the mean log-expression level of the gene g can be expressed as $m_g = \frac{1}{n_{c \neq 0}} \sum_{i:x_{ig} \neq 0} \log x_{ig}$ where $n_{c \neq 0} = \sum_{i=1}^{n_c} I(x_{ij} > 0)$ is the number of cells with non-zero expression of gene g. The next step adopts a heuristic approach from the work of Kobak *et al.* [2] to select 1000 genes by finding a value of b such that there are exactly 1000 genes that exhibit high fraction of zero-expression levels across cells in relation to its mean expression value, which has shown to be able to select biologically relevant genes [8]. Mathematically, this is done by finding a value b such that exactly 1000 genes satisfying the relation $d_g > \exp\left[-\frac{3}{2}(m_g - b)\right] + 0.02$ can be selected. The coefficient $\frac{3}{2}$ and 0.02 are chosen for a good distributional fit [2]. This selected subset of 1000 genes is then kept for the analysis, whereas the others are discarded. Finally, the $\log(1 + x_{ig})$ transformation is applied to the counts of the 1000 selected genes to even out the variance of the larger expression levels. That is, the relative expression difference is considered as opposed to the absolute difference so that, for example, an expression difference from 1 to 5 is considered equal to the difference between 100 and 500.

Author details

References

1. Shannon, C.E.: A mathematical theory of communication. ACM SIGMOBILE mobile computing and communications review **5**(1), 3–55 (2001)
2. Kobak, D., Berens, P.: The art of using t-sne for single-cell transcriptomics. Nature communications **10**(1), 1–14 (2019)
3. Van Der Maaten, L.: Accelerating t-sne using tree-based algorithms. The Journal of Machine Learning Research **15**(1), 3221–3245 (2014)
4. Belkina, A.C., Ciccolella, C.O., Anno, R., Halpert, R., Spidlen, J., Snyder-Cappione, J.E.: Automated optimized parameters for t-distributed stochastic neighbor embedding improve visualization and analysis of large datasets. Nature communications **10**(1), 1–12 (2019)
5. Moon, K.R., van Dijk, D., Wang, Z., Gigante, S., Burkhardt, D.B., Chen, W.S., Yim, K., van den Elzen, A., Hirn, M.J., Coifman, R.R., *et al.*: Visualizing structure and transitions in high-dimensional biological data. Nature biotechnology **37**(12), 1482–1492 (2019)
6. Becht, E., McInnes, L., Healy, J., Dutertre, C.-A., Kwok, I.W., Ng, L.G., Ginhoux, F., Newell, E.W.: Dimensionality reduction for visualizing single-cell data using umap. Nature biotechnology **37**(1), 38–44 (2019)
7. von Luxburg, U.: A tutorial on spectral clustering. Statistics and computing **17**(4), 395–416 (2007)
8. Andrews, T.S., Hemberg, M.: M3drop: dropout-based feature selection for scrnaseq. Bioinformatics **35**(16), 2865–2867 (2019)
