# Supplementary material for: Shape-aware stochastic neighbor embedding for robust data visualisations
Source: BMC Bioinformatics. 2022 Nov 14;23:477. doi: 10.1186/s12859-022-05028-8 (PMC9660178; doi:10.1186/s12859-022-05028-8)
Supplement: Supplementary file 2 — Additional file 2. PDF file containing supporting figures S1–S7. [file 12859_2022_5028_MOESM2_ESM.pdf]

Shape-aware Stochastic Neighbor Embedding for Robust Data Visualisations

Tobias Wängberg[1], Joanna Tyrcha[1*] and Chun-Biu Li[1*]

[1*]Department of Mathematics, Stockholm University, Roslagsvägen 101, Kräftriket, 10691, Stockholm, Sweden.

*Corresponding author(s). E-mail(s): joanna@math.su.se; cbli@math.su.se;

Supplementary figures

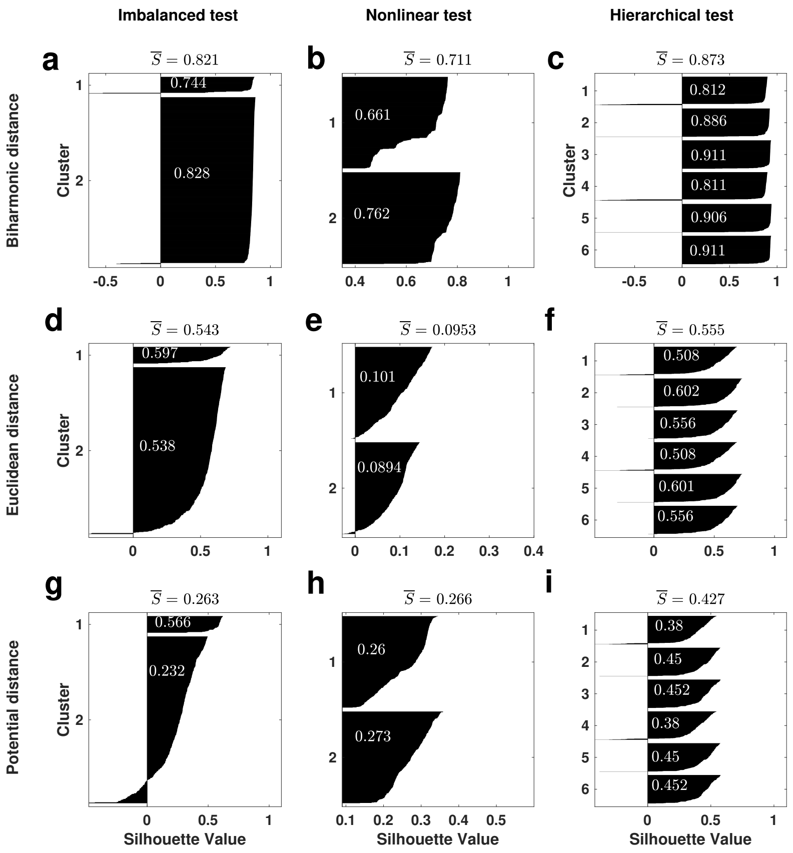

Figure S 1 Silhouette plots comparing BHD, ED and PD for each of the test data sets presented in the main text. This illustrates the advantage of the CTD in terms of clustering because of the increased distance between clusters as well as reduced distance between points within the same cluster. The white number in each black block is the cluster-wise Silhouette score for the corresponding cluster. On top of each plot is the Silhouette score averaged over all points, $\overline{S}$.

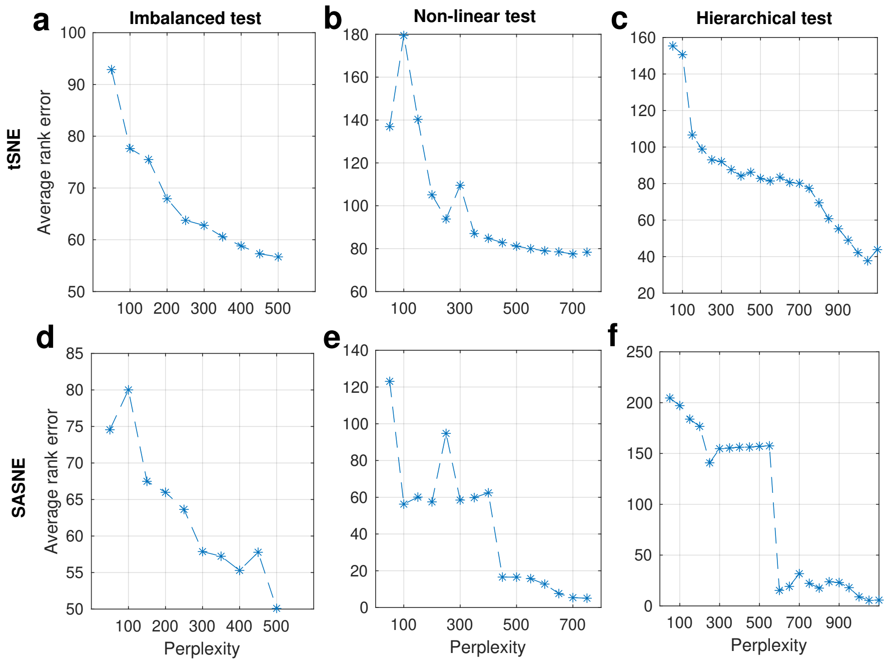

Figure S 2 The figure contains plots of the average rank error $\overline{R}$ for varying perplexity $\mathcal{P}$ values when t-SNE and SASNE are applied to the synthetic data sets presented in the main text. Here we see that the rank error is clearly decreasing when the perplexity increases, showing that larger perplexity values are better for preserving the distance orders between points. **a** $\overline{R}/\mathcal{P}$-curve for t-SNE applied to the imbalanced test. **b** $\overline{R}/\mathcal{P}$-curve for t-SNE applied to the t-SNE the non-linear test.**c** $\overline{R}/\mathcal{P}$-curve for t-SNE applied to the hierarchical test. **d** $\overline{R}/\mathcal{P}$-curve for SASNE applied to imbalanced test. **e** $\overline{R}/\mathcal{P}$-curve for SASNE applied to the non-linear test. **f** $\overline{R}/\mathcal{P}$-curve for SASNE applied to the hierarchical test.

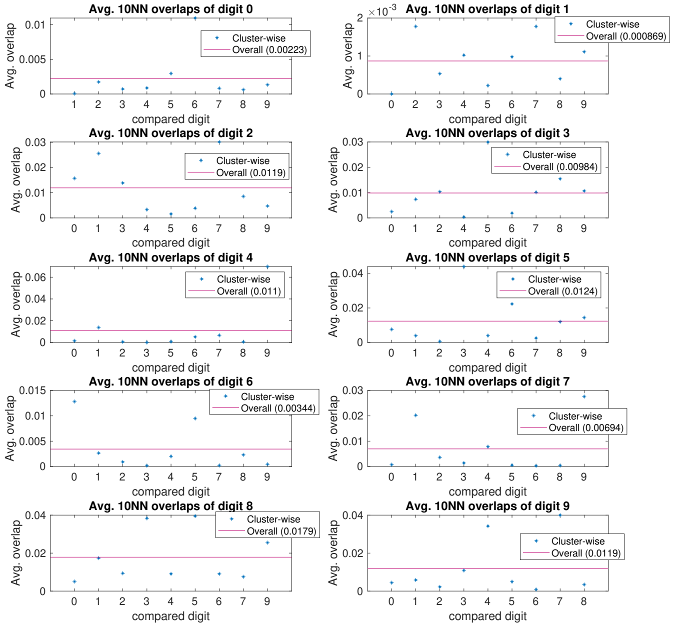

Figure S 3 Overlap between different digit clusters in the MNIST data set analysed in the main text. Each plot shows a diagram where for each digit the average number of unequal digits being among its 10 nearest neighbors (NN) is shown, for each other digit. That is, if each digit where to form distinct clusters, then the overlap would be 0. We see that all clusters overlap to some degree with another cluster. Digits 0, 1 and 6 show the weakest overlap and are therefore relatively distinct compared to the other digits. Digit 2 overlaps overlaps with many digits, mostly with digit 0, 1, 3 and 7. Digit 3 overlaps with digit 5 and 8, and to a lesser degree with digit digits 7 and 9. Digit 4 overlaps strongly with digit 9. Digit 5 overlaps with digit 3, 6, 8 and 9. Digit 6 again quite distinct, but overlaps slightly with digit 0 and digit 5. Digit 7 overlaps with digit 1 and most significantly with digit 9. Digit 8 overlaps strongly with digit 3, 5 and slightly with digit 9. Digit 9 overlaps with digit 4 and 7.

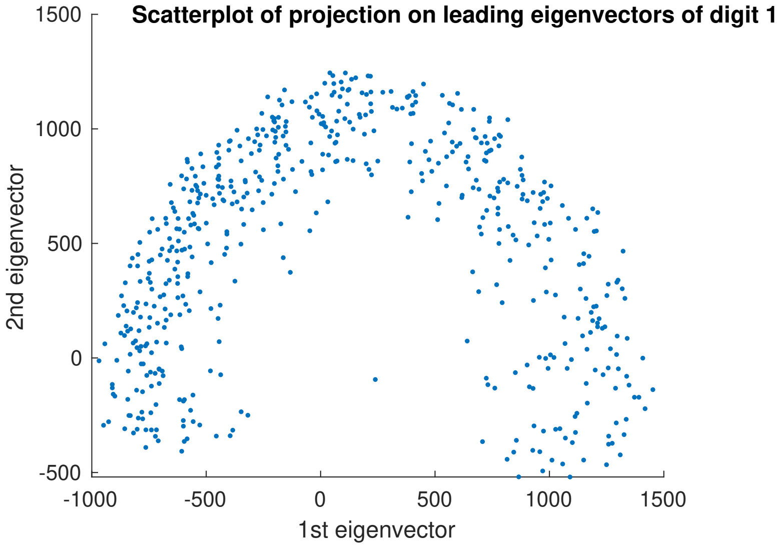

Figure S 4 PCA projection on the 2 leading eigenvectors of the images from the MNIST data set corresponding to digit 1 showing the nonlinear shape of the cluster.

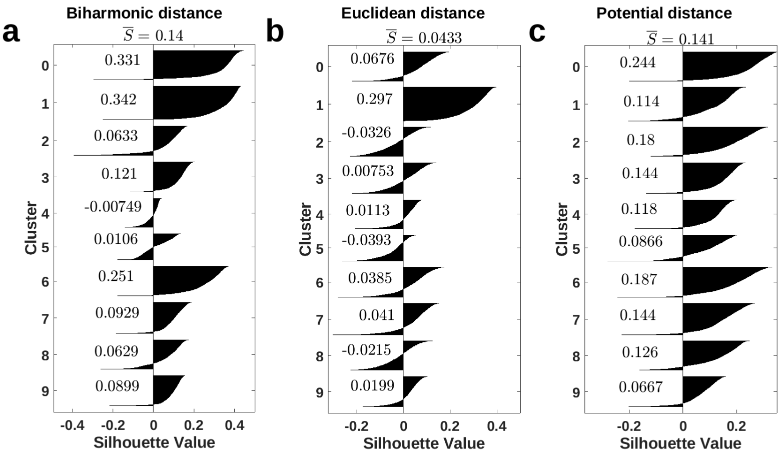

Figure S 5 The figure shows Silhouette plots comparing the Silhouette values based on the CTD and the ED respectively between image vectors in the MNIST data set. The red square contains the per-cluster Silhouette score, as well as the Silhouette coefficient $\overline{S}$. **a** Silhouette plot with silhouette scores according to CTD. **b** Silhouette plot with silhouette scores according to ED. **c** Silhouette plot with silhouette scores according to PD.

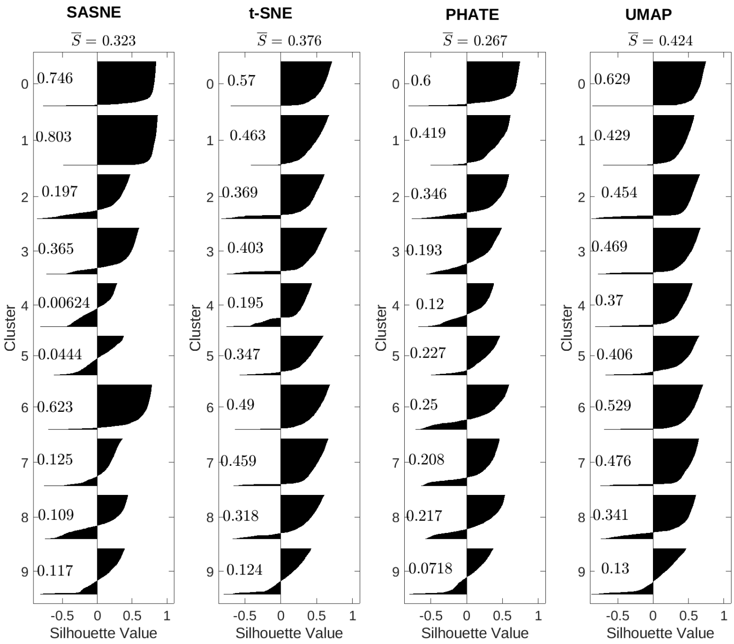

Figure S 6 Pointwise silhouette scores for the SASNE, t-SNE, PHATE and UMAP embeddings of the MNIST data set.

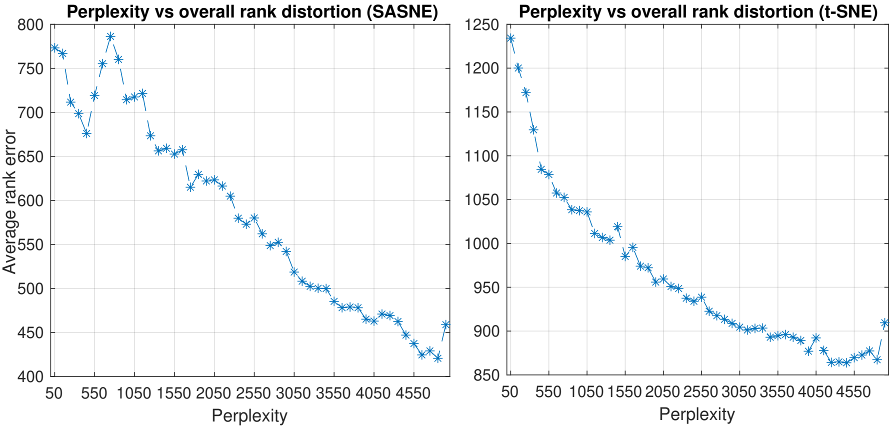

Figure S 7 The figure shows curves of the average rank error $\overline{R}$ against perplexity for t-SNE and SASNE applied to the MNIST data set. Here we observe the trend of lower $\overline{R}$ for larger perplexity values.
